# Accelerating Indonesian COVID-19 vaccination rollout: a critical task amid the second wave

**DOI:** 10.1186/s41182-021-00367-3

**Published:** 2021-09-22

**Authors:** Ryan Rachmad Nugraha, Adriana Viola Miranda, Attaullah Ahmadi, Don Eliseo Lucero-Prisno

**Affiliations:** 1 USAID Health Financing Activity/ThinkWell, Jakarta, Indonesia; 2 Faculty of Medicine, University of Indonesia, Depok, West Java Indonesia; 3 Medical Research Center, Kateb University, Kabul, Afghanistan; 4 Department of Public Health, International School of Medicine, Bishkek, Kyrgyzstan; 5 Department of Global Health and Development, London School of Hygiene and Tropical Medicine, London, UK; 6 Faculty of Management and Development Studies, University of the Philippines (Open University), Los Baños, Laguna, Philippines

**Keywords:** COVID-19, Pandemic, Vaccination, Recommendations, Second wave, Indonesia

## Abstract

Coronavirus disease-19 (COVID-19) has been spreading in every part of the world, putting nations at risk with its pandemic status, including Indonesia. COVID-19 vaccine has been deemed as one of the most effective interventions to date for mitigating the spread and mortality from COVID-19. Responding to the situation, the Government of Indonesia (GOI) has allocated the means necessary to procure and distribute COVID-19 vaccines; placing into consideration the unique context of the country, recently categorized as a middle-income country and archipelagic with a population over 270 million. This article aims to present the challenges associated with the distribution of COVID-19 vaccination as well as recommendations to mitigate them, to ensure a timely and effective COVID-19 vaccination program in Indonesia.

## Background

COVID-19 has ravaged countries around the world [1, 2], wreaking havoc on their healthcare systems [3, 4]. As of June 29, 2021, the total number of COVID-19 cases in Indonesia had reached 2.14 million—the highest in Southeast Asia, with 80% of the cases being the Delta variant [5]. The Government of Indonesia (GOI) has stated that its main strategy to curb the country’s infection rate is to vaccinate at least 70% of the population (181.5 million people) by March 2022 to achieve herd immunity. However, while the program has begun since January, the vaccination rollout has been slow. Currently, only about 5% of Indonesians are fully vaccinated [6]. As the country grapples with the second wave of the pandemic and the emerging Delta variant, improvement of the current COVID-19 vaccination campaign is crucial.

## Indonesian vaccination scheme: overview and challenges

The Indonesian vaccination program is divided into two phases: the first phase, which is supposed to run from January to April 2021, targets 40.2 million healthcare workers, public officials, and the elderly while the second phase (April 2021–March 2022) targets the general public. The vaccines are provided by the government free of charge [7]. In addition, the government also encourages companies to direct their corporate social responsibility funds to cover their employees’ vaccination.

### Vaccine supply problem

The vaccination program mainly uses Sinovac and Sinopharm, two Chinese COVID-19 vaccines bought by the Indonesian government. BioFarma, a government-owned pharmaceutical company, has produced 10–12 million Sinovac doses since January 2021 and is expected to boost its production to over 25 million doses. AstraZeneca is also provided in smaller quantities through Indonesia’s involvement in the COVAX Facility [8]. Pfizer and Novavax vaccines are expected to arrive in July 2021. Indonesia has also begun research projects aimed at producing its own COVID-19 vaccines.

One of the major challenges of the Indonesian vaccination program is the ‘vaccine nationalism’ view currently held by many developed countries. While Indonesia is actively engaging in vaccine diplomacy to secure more vaccines and emphasize its stance against vaccine stockpiling, Indonesia still struggles to procure sufficient vaccines for its population. The first vaccination phase, which was supposed to finish in April, was short on 43.2 million doses. Indonesia has since worked on increasing its vaccine supply. As of July 2021, 99.2 million of vaccine doses, which cover 49.6 million people (27% of the targeted population), have arrived in Indonesia [9]. However, more doses are needed to successfully conduct the second phase of the vaccination program. Many high-income countries have pre-purchase agreements that enable them to procure vaccine doses three to five times their needs. Coupled with global vaccine manufacturing limitations, this means Indonesia has to procure vaccines from a much smaller supply. Indonesia’s COVAX Facility supply has also been impacted by India’s policy to prioritize domestic vaccine needs amid second wave in the country [10].

### Inadequate cold chain system

The Indonesian vaccine cold chain system is established mainly to distribute vaccines for the national childhood immunization program, which only requires a temperature of 2–8 degrees Celsius, with the exception of the polio vaccine (− 15 to 25 °C). However, it is found that even prior to the pandemic, the requirements to put the vaccines in specified temperature and conditions are frequently not followed, rendering the vaccines unsuitable for immunization [11]. During the pandemic, the primary healthcare workers are already struggling with additional workload due to service disruption, which results in more complicated cases and workload. These limitations may hamper effective distribution of vaccines, especially those requiring lower temperature for transport, such as with Pfizer and BioNTech (− 70 °C) vaccine.

The disparity of cold chain facilities is also prominent. While Indonesian primary health centers are already equipped with adequate refrigerators, a recent cold chain capacity assessment reports that as of October 2020 only 12 from 34 provinces have vaccine-compatible storage facilities, most of which are concentrated in the Java Island. Refrigerated containers with moderate mobility are prepared to access other areas, but portable generators are not available in some islands, thus limiting their ability to maintain vaccine quality [12].

### Local anti-vaccination movement

Anti-vaccination groups are getting evident in Indonesia, particularly on COVID-19 vaccines. The latest official survey shows that of 115,000 respondents, 27% expressed hesitation, citing concerns about the vaccine safety, effectiveness, side effects, the country’s health system preparedness as well as issues related to religious beliefs and lack of education. There is growing skepticism shown by a survey that COVID-19 was created by the global elite. Another growing concern on whether the vaccine is permissible according to Islamic law (*halal*), with differing opinions among prominent Islamic organizations. Despite the engagement of Moslem leaders both in national and grassroot levels, the slightest hint of non-Halal compound (i.e., porcine in flu vaccine) might leave people adamant not to vaccinate [13, 14]. In response to the concerns, the Indonesian Ulema Council has issued Halal certifications for Sinovac & AstraZeneca vaccines. However, misinformation regarding the vaccine ingredients remains widespread, especially in social media platforms.

## Recommendations

Improving COVID-19 vaccine rollout in Indonesia requires a comprehensive, multifaceted approach. We recommend the Indonesian government to:

1. Further develop its current vaccine production system & capacity (BioFarma) and allocate more resources for its own vaccine development research projects to enable self-reliance for vaccine supply.
2. Strengthen vaccine diplomacy, especially through dialogues in the COVAX Facility, with focus on ratifying plans for high-income countries to transfer the technology required for vaccine production and share their vaccine excess to LMICs. Purchasing vaccines in proportion to needs should also be encouraged. This is crucial as Indonesia needs to secure more doses from different vaccine manufacturers for its 2022 program. Indonesia’s strategic position as the co-chair of COVAX Advanced Market Commitment Engagement Group (AMC EG) should be utilized to push for more equitable vaccine access worldwide.
3. Encourage provincial governments to allocate funding to improve their cold chain system. The program should focus on provinces that lack vaccine-compatible systems, especially those outside Java Island.

Indonesian MoH should:

1. Collaborate actively with provincial governments to monitor cold chain system quality and needs in their respective locations. The Indonesian Ministry of Finance has allocated 467.45 billion IDR (approx. USD 33,200) to procure medical equipment for distribution, storage, and vaccination process.
2. Collaborate with local governments for the implementation of the COVID-19 vaccination program, including for local funding allocation. Indonesia follows a decentralization policy, in which the local governments have autonomy over their regions. During the COVID-19 lockdown, the Indonesian local governments had implemented different measures in reaction to the national government policies, with varying results: from a successful limitation of the virus spread to less ideal results. This led the Indonesian president to urge the local governments to firstly discuss their policies with the national government. Similar collaborations are needed to ensure the acceleration of the vaccination program.
3. Engage organizations who show resistance toward COVID-19 vaccines through discussions and health promotion, as to influencing those within the organization who express and spread hesitancy.

Indonesian local governments should:

1. Collaborate with national governments, including by allocating more funding, to enable the acceleration of the vaccination program.

## Conclusion

To succeed, the Indonesian COVID-19 vaccination program needs to address challenges regarding the vaccine cold chain and distribution system, as well as its citizens’ acceptance of the vaccine. Although several efforts have been put into action to mitigate these challenges, additional measures are needed to ensure the program reaches its intended target of 70% national coverage.

## Data Availability

Not applicable.

## Abbreviations

COVID-19: Novel coronavirus disease
AMCEG: Advanced Market Commitment Engagement Group
LMICs: Low- and middle-income countries
IDR: Indonesia Rupiah
